# Mechanical Small Bowel Obstruction Due to Gallstone Ileus: Diagnostic Challenges and Surgical Management

**DOI:** 10.7759/cureus.44153

**Published:** 2023-08-26

**Authors:** Mena Louis, Brian Gibson, Louise Jones, Hardeep Singh

**Affiliations:** 1 General Surgery, Northeast Georgia Medical Center Gainesville, Gainesville, USA; 2 Trauma and Acute Care Surgery, Northeast Georgia Medical Center Gainesville, Gainesville, USA; 3 Research, Northeast Georgia Medical Center Gainesville, Gainesville, USA; 4 Research, Northeast Georgia Medical Center Braselton, Gainesville, USA

**Keywords:** rigler’s triad, cholecystoduodenal, enterolithotomy, pneumobilia, ileus, gallstone

## Abstract

Gallstone ileus is a true mechanical intestinal obstruction. It is caused by gallstone impaction in the gastrointestinal (GI) tract after eroding and passing through a bilioenteric fistula. Gallstones are frequently impacted in the terminal ileum. Computed tomography (CT) imaging is diagnostic and shows specific findings of dilated small bowel loops suggesting small bowel obstruction, pneumobilia, and impacted gallstone in the small bowel. Favorable outcome is achieved by having strong clinical suspicion, timely diagnosis, preoperative resuscitation, and early surgical intervention. The three available surgical procedures to relieve gallstone ileus are entrolithotomy alone; one-stage procedure of enterolithotomy, cholecystectomy, and fistula closure; or two-stage procedure of enterolithotomy followed by cholecystectomy. This article outlines the clinical presentation, diagnosis, resuscitation, and different surgical interventions of patients with gallstone ileus.

## Introduction

Gallstone ileus is a rare cause of mechanical small bowel obstruction with an incidence of approximately 1% of all small bowel obstructions [1]. It is caused by the impaction of a gallstone in the small bowel, after eroding and passing through a bilioenteric fistula typically formed between the gallbladder and the first or second part of the duodenum [2]. This disease is mislabeled as ileus; it is a true mechanical small bowel obstruction commonly seen in elderly female patients with several associated comorbidities [2,3]. It accounts for less than 1% of mechanical small bowel obstruction [1,3].

Gallstone ileus is usually preceded by acute cholecystitis and subsequent development of inflammatory adhesions with surrounding portions of the gastrointestinal (GI) tract [1,2]. Spontaneous biliary enteric or cholecystoenteric fistula is due to ischemia of the gallbladder wall created by an obstructing gallstone [2]. Chronic inflammation and ischemic necrosis of the gallbladder wall cause a fistula tract formation between the gallbladder and adjacent duodenum (cholecystoduodenal), transverse colon (cholecystocolic), or stomach (cholecystogastric) [1-5]. The fistula most commonly develops between the gallbladder and duodenum (first and second parts) due to anatomical proximity [6,7]. The progression to a fistula is variable and depends on several factors, such as the presence of gallstones, the severity and persistence of inflammation, the effectiveness of treatment, and individual patient factors, such as age, comorbidities, and overall health. It may take weeks to months of chronic inflammation to lead to the formation of a fistula. Specific time frames are challenging to define, as the process can be highly variable among individuals.

The gallstone that may pass to the small intestine is commonly impacted in the terminal ileum and ileocecal valve due to its narrow lumen and less peristaltic activity [8]. Computed tomography (CT) imaging is diagnostic, and this is managed by surgical interventions to remove the stone and relieve the obstruction. The surgical interventions are enterolithotomy, one-stage procedure, or two-stage procedure [1,2]. The enterolithotomy is recommended as it has lower morbidity and mortality [1].

In this case report, the aim is to provide an in-depth exploration of gallstone ileus, focusing on the underlying pathophysiology of cholecystoenteric fistula formation. The report covers various aspects, including the clinical presentation, diagnostic methodologies, workup, preoperative resuscitation strategies, and tailored surgical interventions for managing gallstone ileus. The comprehensive approach is designed to educate and inform on all facets of the condition, from initial symptoms to surgical management.

## Case presentation

An 80-year-old male with a history of hypertension and no history of biliary manifestation presented to the emergency department with a five-day history of abdominal pain with distension, nausea, and vomiting. Upon physical examination, the patient's abdomen was observed to be significantly distended, exhibiting tympanitic sounds upon percussion, and demonstrated diffuse tenderness upon palpation. The initial laboratory results, obtained in the emergency department, are delineated in Table 1.

**Table 1:** Pre-operative laboratory results. BUN, blood urea nitrogen; WBC, white blood cell

Labs	Value	Reference range
Sodium	131	135-148 mmol/L
Potassium	3.9	3.5-5.2 mmol/L
Chloride	92	100-110 mmol/L
CO_2_	33	21-32 mmol/L
BUN	42	5-23 mg/dL
Glucose	155	65-99 mg/dL
Creatinine	1.53	0.8-1.3 mg/dL
WBC	12.8	4.8-10.8 K/ul
Hemoglobin	15.4	14-18 g/dL
Hematocrit	44.7	42-52%
Platelets	404	130-400 K/ul

**Figure 1:**
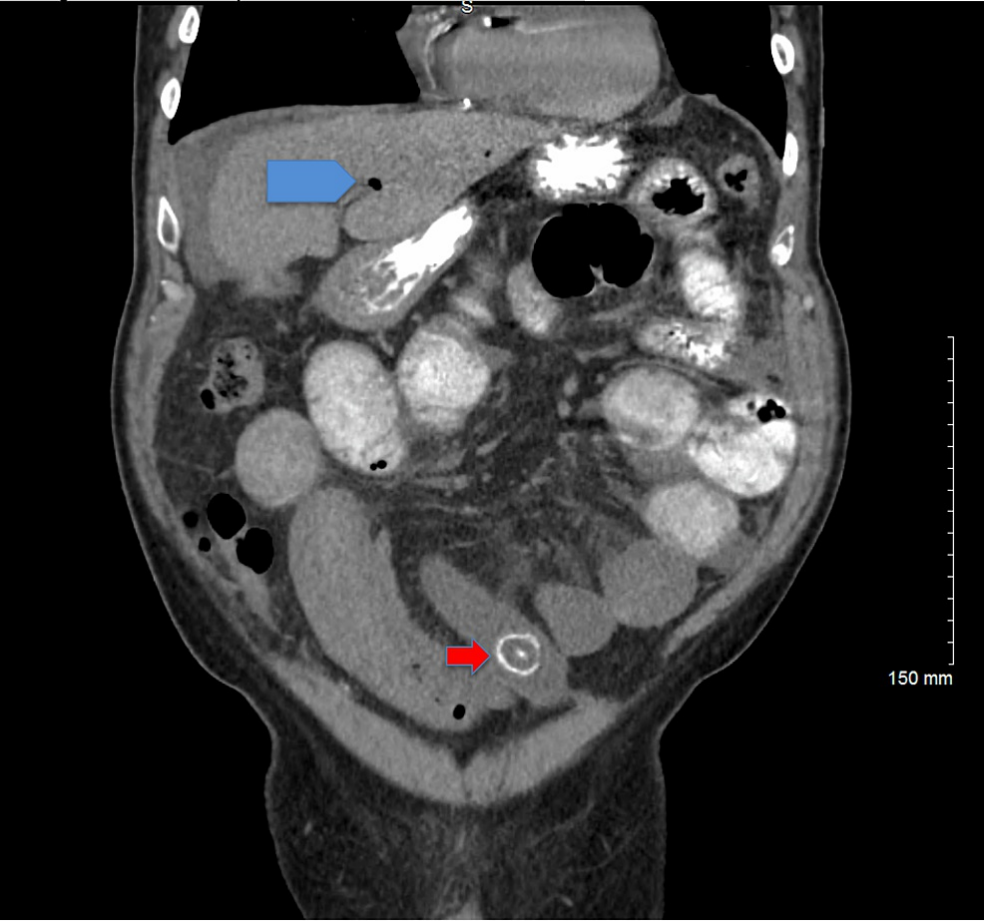
Coronal CT of abdomen and pelvis showing dilated small bowel loops, pneumobilia (blue pentagon), and impacted gallstone in the small bowel (red arrow).

**Figure 2:**
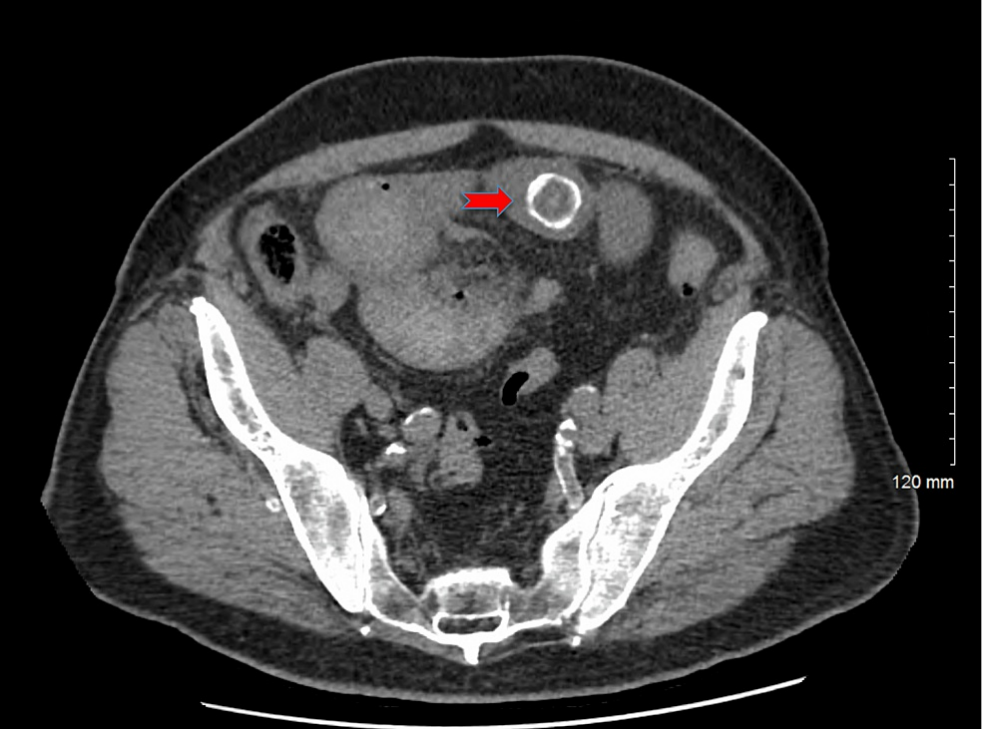
Axial CT highlighting an impacted gallstone in jejunum (red notched arrow).

**Figure 3:**
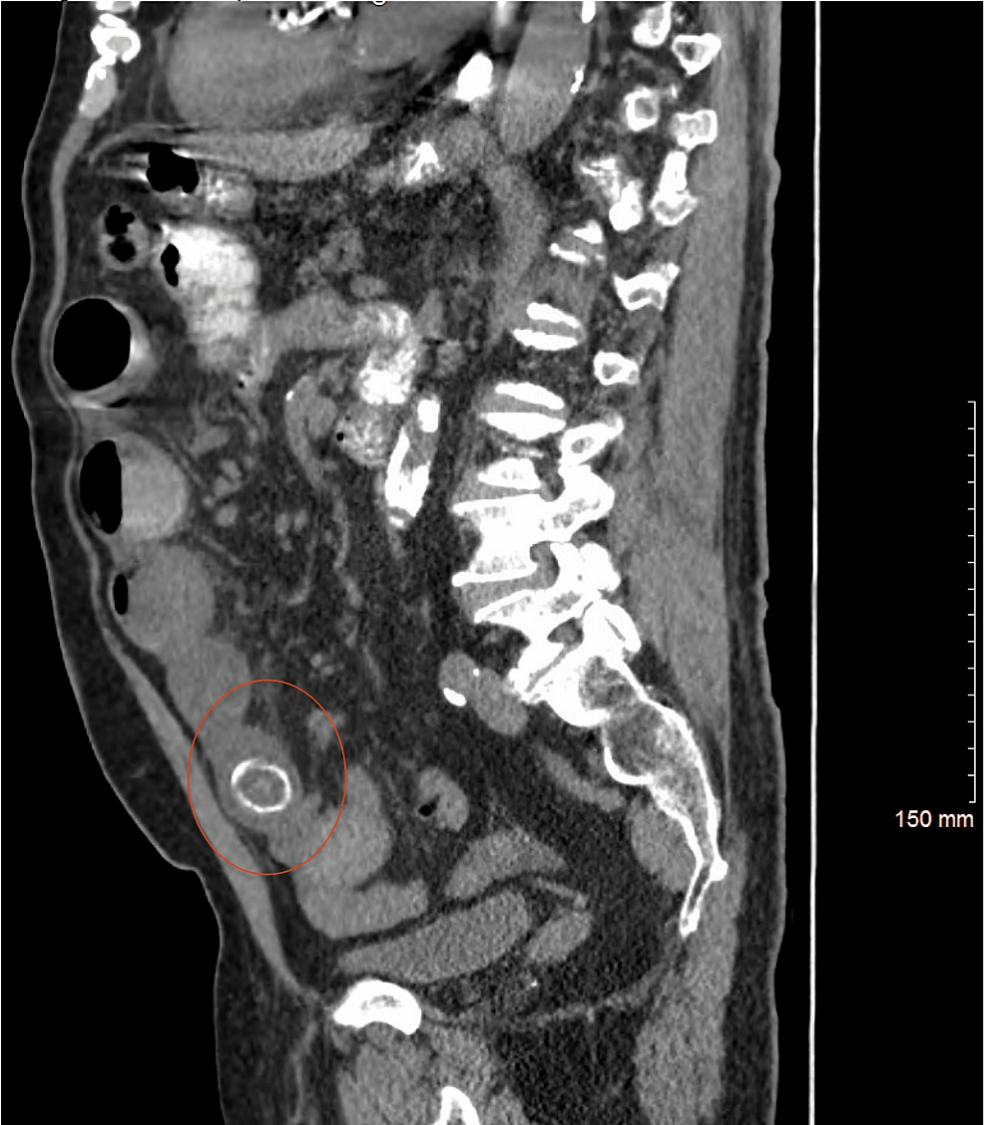
Sagittal CT abdomen and pelvis showing the dilated small bowel loops and impacted gallstone in the jejunum (red circle).

A CT scan of the abdomen and pelvis with intravenous (IV) contrast was obtained, and the findings were indicative of gallstone ileus. In addition, the scan revealed a fistula between the second part of the duodenum and the gallbladder. Minimal ascites were also noted, which were presumed to be reactive in nature (as illustrated in Figures 1, 2, 3). 

Surgical operation: laparoscopy and removal of the impacted stone via enterolithotomy

**Figure 4:**
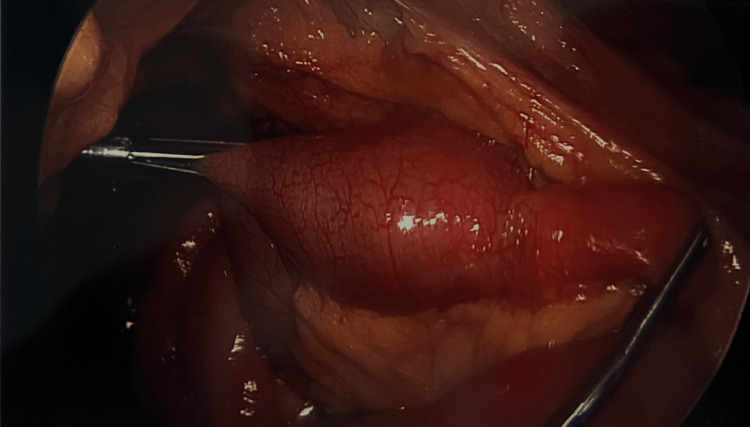
Laparoscopic view of the impacted gallstone in the jejunum.

**Figure 5:**
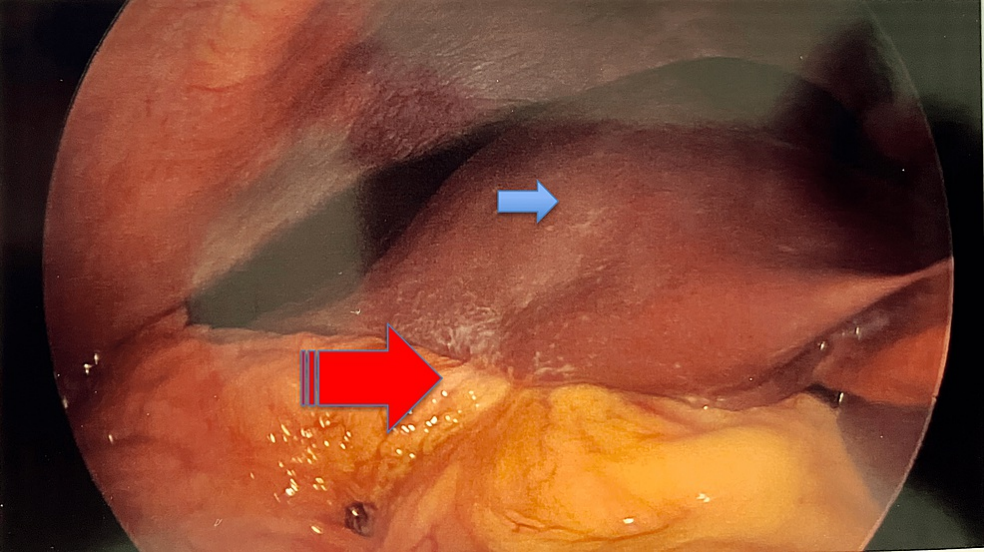
Laparoscopic view of the liver (blue arrow) showing significant inflammatory adhesions with the omentum and duodenum (striped red arrow).

**Figure 6:**
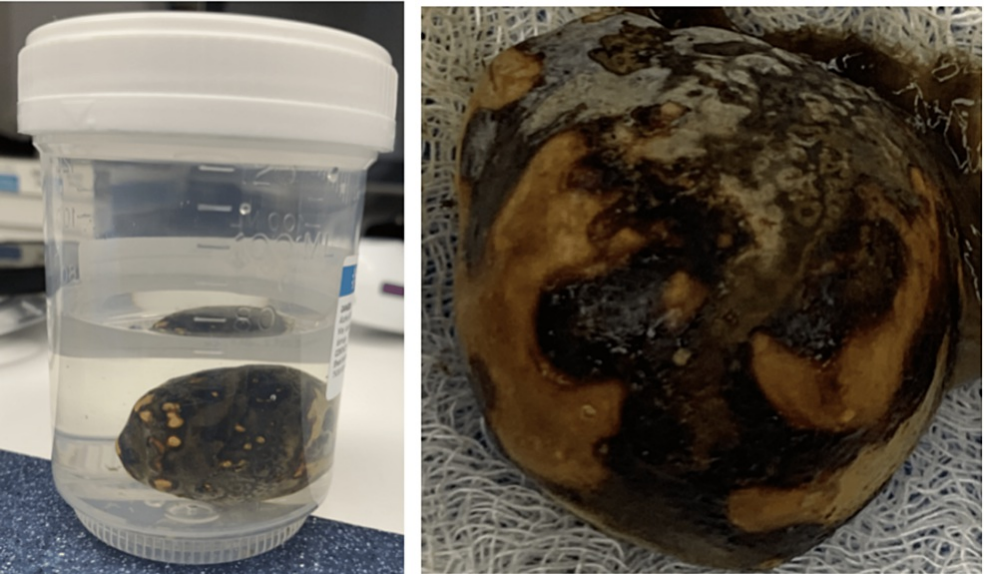
Extracted gallstone measuring 4 x 4 cm.

The patient was placed in the supine position under general anesthesia. An upper midline incision was made, the GelPort laparoscopic system was placed, and the abdomen was insufflated. Subsequently, two additional 5 mm trocars were placed in the suprapubic and left upper quadrant. The small bowel was found to be distended proximally and decompressed distally. At the distal jejunum, there was a palpable mobile mass within the bowel lumen that was consistent with preoperative imaging (Figure 4). This part of the small bowel was brought out through the midline incision. A longitudinal incision was made in the antimesenteric border of the small bowel overlying the impacted gallstone. Gallstone was extracted and the enterotomy was then closed transversely with an inner Vicryl suture and external layer of interrupted silk. Manipulation of the cholecystoduodenal fistula was not attempted due to severe inflammatory adhesions (Figure 5). The bowel was then returned to the abdomen, and the abdomen was closed. The extracted gallstone measuring 4 x 4 cm is shown in Figure 6.

Postoperatively, the patient had an uneventful postoperative course and regained bowel function, and the nasogastric tube was removed on postoperative day 2. Then, a clear liquid diet was given, which was advanced to a regular diet on postoperative day 3. The patient was discharged home on postoperative day 4.

## Discussion

In 1654, Thomas Bartholin described the first case of gallstone ileus in a necropsy study [9,10]. Gallstone ileus is a disease of the elderly and frail with associated multiple comorbidities [11]. It is an intraluminal intestinal obstruction caused by a large gallstone that enters the intestinal lumen through a fistula usually between the gallbladder and duodenum, but the stomach, small bowel, and transverse colon can also be involved [3,12].

Gallstone ileus is frequently preceded by a history of cholecystitis or biliary symptoms of upper abdominal pain, nausea, vomiting, and dyspepsia [1,2]. Symptoms and signs of gallstone ileus are nonspecific, and patients frequently experience intermittent cramping abdominal pain, distension, constipation, nausea, and vomiting [13,14]. As the gallstone advances in the intestine, "tumbling" can lead to intermittent partial small intestinal obstruction and subsequent relief of the obstruction, until the stone is impacted in a narrow part of the small intestine and causes complete intestinal obstruction [8,13]. This "tumbling phenomenon" explains why most patients with gallstone ileus do not seek medical attention and present late with complete intestinal obstruction [1,11]. 

If the gallstone is impacted in the pylorus, it can lead to gastric outlet obstruction, also known as "Bouveret’s syndrome," that may be preceded by symptoms of upper GI bleeding due to stone erosion of the duodenum [1,6,12]. 

On physical examination, patients are frequently acutely ill and present with signs of dehydration [6,15]. The physical examination is non-specific, but patients often have abdominal distension and tenderness with associated high-pitched bowel sounds [5,6]. If intestinal perforation occurs, patients present with signs of peritonitis and fever [11,13].

The classic radiologic sign of gallstone ileus is Rigler’s triad, which includes dilated small bowel loops, pneumobilia, and impacted gallstones in the small bowel [11,15]. This triad can be seen on a plain abdominal X-ray film. CT abdomen and pelvis is the diagnostic study of choice with a high sensitivity and specificity [15]. The presence of two of the three Rigler's triad signs is considered diagnostic [15].

Pneumobilia (presence of air within the biliary tree) is usually due to the abnormal communication between the bowel and biliary tree (biliary-enteric fistula); other causes of pneumobilia include an infection by a gas-forming organism [4,14,15]. Sphincterotomy after endoscopic retrograde cholangiopancreatography (ERCP) is also a common cause of pneumobilia [15]. Other causes of pneumobilia include cholangitis, liver abscess, and emphysematous cholecystitis [10,11]. 

The bowel obstruction can be complete or partial. In small bowel obstruction, gas and secretions accumulate proximal to the obstruction leading to dilation of the bowel [14]. Initially, the small bowel increases peristalsis to overcome the obstruction. The dilation of the small bowel leads to nausea and colicky abdominal pain with emesis [11]. The bowel sounds are high pitched initially, but later, the small bowel motility and bowel sounds decrease [7]. 

Dehydration is common in small bowel obstruction and is due to frequent episodes of emesis that may also lead to electrolyte abnormalities and hyperchloremic hypokalemic metabolic alkalosis [4,11,16]. The accumulation of fluid and gas in the bowel lumen proximal to the obstruction results in stasis and bacterial overgrowth [11,17]. This progressive distension can lead to increased intraluminal hydrostatic pressure, which causes transudative loss of fluids and electrolytes into the bowel wall and peritoneal cavity [1,4]. This process is called third spacing, and it contributes to dehydration [14].

Evaluation, workup, and resuscitation 

Assess Vital Signs and Hydration Status

IV access, a nasogastric tube, and a Foley catheter should be placed immediately [11,18]. Resuscitation is initiated with normal saline or lactated Ringer solutions [16,17]. Urine output is used to predict adequate resuscitation and should be kept at least 0.5 ml/kg/hr and followed by 40 mEq/L supplemental potassium chloride administration [11,16,18]. During abdominal examination, high-pitched bowel sounds are suggestive of an obstruction. The absence of bowel sounds is noticed in the case of ileus or intestinal paralysis but can also occur after intestinal fatigue after long-standing bowel obstruction [11].

Investigation starts with an abdominal X-ray series, followed by CT of the abdomen and pelvis with IV and oral contrast, which has a higher specificity and can identify the level and severity of obstruction [2,11,15]. Bowel obstruction is identified by dilated loops of small bowel with multiple air-fluid levels [17]. The presence of pneumoperitoneum and pneumatosis (gas within the wall of the intestine) may signify ischemic bowel and necrosis. Portal venous gas is a late presentation of pneumatosis with air passing through the portal venous circulation to the periphery of the liver [18]. 

Complete blood count (CBC), serum electrolytes, serum creatinine, coagulation profile, and serum lactate can be used to determine the severity of obstruction and guide resuscitation [11]. Gallstone Ileus as a cause of bowel obstruction is usually seen extending uniformly throughout the stomach and the small and large bowels [18,19]. Patients should be evaluated for other comorbid conditions, including cardiopulmonary and metabolic diseases, that would affect the prognosis [7,11,17]. Patients with gallstone ileus are managed initially with nasogastric decompression, fluid resuscitation, and electrolyte repletion [18,19]. 

Gallstone ileus requires emergency surgical intervention to extract the stone and relieve the obstruction [9,16]. The surgical procedures to relieve the bowel obstruction are either enterolithotomy alone; one-stage procedure of enterolithotomy, cholecystectomy, and fistula closure; or two-stage procedure of enterolithotomy followed by cholecystectomy [1,9,16]. It is recommended to palpate for, identify, and extract any additional intraluminal stones impacted in the bowel, which is a common cause of recurrence [13,16]. The common locations for stones are the distal ileum, followed by the jejunum and gastric outlet [1,13]. 

Enterolithotomy alone: The bowel obstruction is relieved through a proximal longitudinal enterotomy on the antimesenteric border to extract the stone and the enterotomy is closed in a transverse fashion to avoid narrowing [1,5,9]. If bowel ischemia, perforation, or stenosis is identified during the procedure, bowel resection may be necessary [16]. Although enterolithotomy alone is associated with lower morbidity and mortality compared to one- or two-stage procedures, its main drawback is unaddressed biliary fistula, which may cause a recurrence [1,6]. Spontaneous closure of the biliary enteric fistula is frequently reported [10,13]. Thus, closure of the fistula is not necessary during enterolithotomy [3,9,10].

One-stage procedure of enterolithotomy, cholecystectomy, and fistula closure: It is usually performed for highly selected patients who present with acute cholecystitis, gangrenous gallbladder, or residual gallstones [4,9,18]. It is associated with higher morbidity and mortality when compared with enterolithotomy alone [16].

Two-stage procedure: It includes enterolithotomy that is subsequently followed in four to six weeks by cholecystectomy and closure of the biliary enteric fistula tract [1,6,16]. 

There is a high rate of mortality with performing a one-stage procedure of enterolithotomy, cholecystectomy, and closing the biliary-enteric fistula in the presence of acute inflammation associated with gallstone ileus [8,13]. If the fistula tract is left, the rate of recurrence of gallstone ileus is less than 5% [2,3,9]. Therefore, enterolithotomy and leaving the biliary enteric fistula is advocated, especially in elderly and frail patients with multiple comorbidities [1,14,16].

## Conclusions

Gallstone ileus, a rare cholelithiasis complication, mainly affects the elderly with underlying comorbidities. The diagnosis relies on small bowel obstruction and CT findings but can be delayed due to the gallstone's erratic movement, causing intermittent symptoms. Patients often present with fluid and metabolic imbalances, complicating existing conditions. Favorable outcomes are achieved through a strong clinical suspicion, timely diagnosis, preoperative resuscitation, and early surgery. Enterolithotomy alone is advised for the elderly, while a one-stage procedure is reserved for fit and stable patients with additional complications.

## References

[1] Nuño-Guzmán CM, Marín-Contreras ME, Figueroa-Sánchez M, Corona JL (2016). Gallstone ileus, clinical presentation, diagnostic and treatment approach. World J Gastrointest Surg.

[2] Smith D, Amiri F, Denning D (2023). Gallstone ileus: a case report in a 74-year-old male. Am Surg.

[3] Tsang CF (2021). A rare case of gallstone ileus-the unanswered question. J Surg Case Rep.

[4] Martin F (1912). Intestinal obstruction due to gall-stones: with report of three successful cases. Ann Surg.

[5] Kasahara Y, Umemura H, Shiraha S, Kuyama T, Sakata K, Kubota H (1980). Gallstone ileus. Review of 112 patients in the Japanese literature. Am J Surg.

[6] Inukai K (2019). Gallstone ileus: a review. BMJ Open Gastroenterol.

[7] Ghimire P, Maharjan S (2023). Adhesive small bowel obstruction: a review. JNMA J Nepal Med Assoc.

[8] Chang L, Chang M, Chang HM, Chang AI, Chang F (2018). Clinical and radiological diagnosis of gallstone ileus: a mini review. Emerg Radiol.

[9] Neary PM, Dowdall JF Evolution of entero-biliary fistula following gallstone ileus management. BMJ Case Rep. Sep.

[10] Turner AR, Sharma B, Mukherjee S (2023). Gallstone Ileus. StatPearls [Internet].

[11] Amara Y, Leppaniemi A, Catena F (2021). Diagnosis and management of small bowel obstruction in virgin abdomen: a WSES position paper. World J Emerg Surg.

[12] Ayantunde AA, Agrawal A (2007). Gallstone ileus: diagnosis and management. World J Surg.

[13] Abou-Saif A, Al-Kawas FH (2002). Complications of gallstone disease: Mirizzi syndrome, cholecystocholedochal fistula, and gallstone ileus. Am J Gastroenterol.

[14] Beuran M, Ivanov I, Venter MD (2010). Gallstone ileus--clinical and therapeutic aspects. J Med Life.

[15] RO FA, CA R (1958). Gallstone intestinal obstruction. Calif Med.

[16] Roothans D, Anguille S (2013). Rigler triad in gallstone ileus. CMAJ.

[17] Jakubauskas M, Luksaite R, Sileikis A, Strupas K, Poskus T (2019). Gallstone ileus: management and clinical outcomes. Medicina (Kaunas).

[18] Klingbeil KD, Wu JX, Osuna-Garcia A, Livingston EH (2023). Management of small bowel obstruction and systematic review of treatment without nasogastric tube decompression. Surg Open Sci.

[19] Maung AA, Johnson DC, Piper GL (2012). Evaluation and management of small-bowel obstruction: an Eastern Association for the Surgery of Trauma practice management guideline. J Trauma Acute Care Surg.

